# Addressing COVID-19 in Afghanistan: What are the efforts and challenges?

**DOI:** 10.7189/jogh.10.020341

**Published:** 2020-12

**Authors:** Don Eliseo Lucero-Prisno, Attaullah Ahmadi, Mohammad Yasir Essar, Xu Lin, Yusuff Adebayo Adebisi

**Affiliations:** 1 Department of Global Health and Development, London School of Hygiene and Tropical Medicine, UK; 2 Kabul University of Medical Sciences, Kabul, Afghanistan; 3 Department of Thoracic Surgery, The First Affiliated Hospital, School of Medicine, Zhejiang University, Hangzhou, Zhejiang, PR China; 4 Faculty of Pharmacy, University of Ibadan, Ibadan, Nigeria

COVID-19 pandemic is a global public health threat [1]. The first case of COVID-19 was reported in the city of Herat in the western part of Afghanistan on 24 February 2020 [2]. This place borders with Iran where many returnees have seeded new cases in the country [3]. As of 11 June 2020, the virus has spread to all 34 provinces with 22 890 confirmed cases and 426 deaths. 3326 patients have recovered so far [4]. Kabul is more highly affected than any other province with 9140 confirmed cases; followed by Herat, Balkh, Nangarhar and Kandahar [5].

When the signs of first cases were reported, the government declared an emergency response to avoid the further spread of the virus. This response was not enough to curb the spread until the time some restrictions in the country were lifted, which followed surges in the cases. At present, the country’s indicators are very dire. This article aims at providing a critical commentary on the current efforts against COVID-19 pandemic and the challenges facing its responses in Afghanistan.

## Efforts

During the early days of the epidemic in the country, the Ministry of Public Health of Afghanistan had explicitly asserted that people should strictly follow the guidance provided by the ministry otherwise 80% percent of the whole population will be infected by the virus which may cause many deaths [6]. Thus, the government initiated measures in order to curb the spread of the virus. First, it started to launch the screening program for those entering through the country’s porous borders and airports. Those exhibiting the symptoms were carried by ambulances to the isolation centers designated by the Ministry of Public Health, and those without any symptoms were recommended to quarantine at their homes for 14 days [6]. According to the World Health Organization (WHO) [7], as of 3 June 2020, 324 464 people have been screened for symptoms. Furthermore, programs on raising awareness in the population in preventing the spread of the virus were conducted. Over 1 092 889 people have benefited from WASH assistance and hygiene promotion programs [4]. As of now, a total number of 2000 of beds for ICU and isolation centers are operational with a 100-bed hospital in Herat and a 300-bed isolation center at Darulaman Palace including two governmental university dormitories [5,7].

Initially, only one testing laboratory was functional in Kabul. The capacity has been increased to 11 testing laboratories – five in Kabul and one each in Herat, Balkh, Kandahar, Nangarhar, Paktia and Kunduz provinces [5]. Lockdown was first imposed in Herat then subsequently in all major provinces including Kabul. Schools and universities were closed, and restrictions on mass gatherings were imposed. Moreover, restrictions on highways leading to the provincial centers were also included in the plan [6]. Based on that, patients and their immediate family members, returnees, military personnel, and food suppliers were the only ones allowed to use transport on the major highways. The daily routine of governmental and private hospitals was recommended to continue on a daily basis [6]. On 6 June 2020, the government extended the nationwide lockdown for three more months due to the deterioration of the situation. Based on this, provincial authorities could set their own restrictions and regulations according to the severity of the epidemic in their provinces [4].

## Challenges

Afghanistan’s fragile health system continues to face many challenges. The protracted conflict in the country has affected the delivery of health care services. Health workers have frequently been targeted and their activities have been discouraged. According to The United Nations Assistance Mission in Afghanistan (UNAMA) [8], a total of 75 incidents in 2019, compared to 65 in 2018, occurred on health care workers and health facilities. These incidents included direct attacks which claimed lives in some cases as well as missing 24 000 hours of health care services and 41 000 consultations. Of the total 75 incidents, 57 were claimed to have been carried out by anti-government elements including 53 by the Taliban and 17 incidents by other forces. On 12 May 2020, the attack on Dasht-e-Barchi maternity hospital, a governmental MSF (Doctors Without Borders)-assisted hospital which took lives of mothers, babies and health care workers attests to the vulnerability of health care services [9].

Despite devising all aforementioned efforts and strategies, Afghanistan is not fully successful in tussling the virus. The number of new cases in the country is continuously surging with a test positivity rate of 43 percent [4]. The aid to the health sector from the World Bank, foreign countries and foundations has provided some breather but the country still remains ill-equipped to deal appropriately with the progressing predicament. At the present time, not enough personal protective equipment (PPE) are available, thus, threatening the lives of health workers. According to the WHO [7], of the required 425 000 PPE, only 15 000 are available. This shortage directly accounts to more than five percent of total confirmed cases among health workers in the country.

Insufficient number of health workers and testing capacity, high rate of illiteracy and the adverse economic situation, are other massive challenges in tackling the virus. They continue to contribute to the surges. According to WHO, there are 9.4 skilled health professionals and 1.9 disproportionately distributed physicians per 10 000 individuals [2]. As of June 11, 2020, only 52 546 individuals out of 37.6 million population have been tested [4]. This implies that the true number of infected cases and deaths are considerably underreported. The Ministry of Public Health of Afghanistan states that they are able to test only 2000 samples out of 10 000 to 20 000 received daily [10]. Underreporting the real number of cases to the general public has made the population oblivious in seriously following the restrictions as they did during the early days of the crisis.

**Figure:**
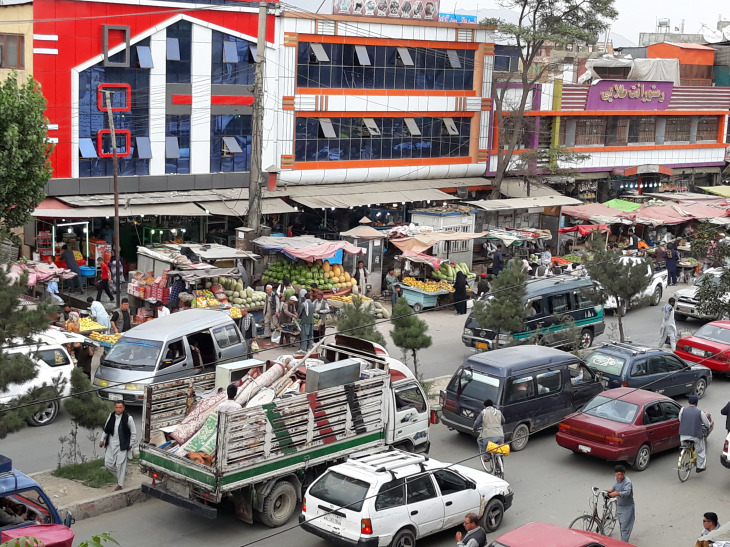
Photo: People busy with their daily routine amid COVID-19 pandemic in the city of Kabul (owned by one of the co-authors, used with permission).

Since COVID-19 contagion started, Afghanistan’s health care system has continuously been facing countless challenges. The situation remains fragile due to many problems the country has been facing for a long period of time. The spread of the virus throughout the country has disproportionately affected the health system in ways where the burden continues to be a heavy load. The government has no other programs to help those who have lost their jobs, except providing needy families with bread across the country and waiving water and power bills for select groups in Kabul since the start of the lockdown. These hardly addressed the needs of the people and hence they were forced to leave their homes searching for jobs and other provisions.

In addition, influx of hundreds of thousands of migrants and refugees from Iran, Pakistan, Turkey, and some European Union countries back to Afghanistan has aggravated the situation. Ninety percent of the returnees had left the country due to economic reasons including unemployment [11]. In spite of the unfavorable situation with viral spread, the government, eventually, ratified the plan of lifting restrictions in order to mitigate the worsening economic costs. This policy allowed some businesses, such as money exchange markets and shops, to operate based on a schedule approved by the government as recommended by the Ministry of Public Health. An even-odd license plate policy was also implemented allowing vehicles to operate alternately based on even and odd days thus lessening the number of transports on the road [12].

## CONCLUSION

Afghanistan’s current outbreak status clearly shows that the country is heading towards the calamity that experts had warned. Although some level of intensity has been reduced through the help of international donors, still there are many gaps that need to be addressed as soon as possible. A high level of intervention is needed from the wealthy nations and other donors to avoid further catastrophe.
